# Renal abscess with bacteremia caused by extended-spectrum β-lactamase-producing *Escherichia coli*: a case report

**DOI:** 10.1186/s12887-020-02366-5

**Published:** 2020-10-06

**Authors:** Hiroki Kitaoka, Jun Inatomi, Hayato Chikai, Keiko Watanabe, Tadayuki Kumagai, Ayako Masui, Nobutaka Shimizu

**Affiliations:** 1 Department of Pediatrics, Yaizu City Hospital, 1000 Doubara, Yaizu-shi, 425-8505 Shizuoka Japan; 2 Department of Pediatrics, Teikyo University Mizonokuchi Hospital, Kawasaki-shi, Kanagawa Japan

**Keywords:** Urinary tract infection, Renal abscess, Extended-spectrum β-lactamase, Pediatrics, Case report

## Abstract

**Background:**

Renal abscess in children is a rare and severe form of infectious kidney disease that is responsible for several serious complications. In this report, we describe a previously healthy 5-year-old girl with a renal abscess caused by extended-spectrum β-lactamase (ESBL)-producing *Escherichia coli (E. coli)*, which led to bacteremia and renal scarring.

**Case presentation:**

The patient presented to our department with high fever, headache, vomiting for 2 days and high inflammatory response. We diagnosed her with a urinary tract infection and initiated treatment with ampicillin and cefotaxime. Gram-negative bacilli bacteremia was noted on day 3. On day 4, her fever persisted, and a computed tomography (CT) scan revealed a renal abscess in the left kidney. After identifying the bacteria as ESBL-producing *E. coli* from the blood culture, we switched to the antibiotic meropenem and continued treatment for 3 weeks. The renal abscess was not drained. Although the renal abscess was successfully treated and it disappeared, a low-density area remained in same lesion on subsequent CT scans and a dimercaptosuccinic acid renal scan performed 4 months after onset revealed renal scarring.

**Conclusion:**

Given the increasing prevalence of ESBL-producing microorganisms, clinicians should be aware of the possibility of renal abscesses caused by community-acquired ESBL-producing organisms even in previously healthy children. Once a renal abscess is suspected, early diagnosis and management are important for reducing the risk of life-threating complications and renal scarring.

## Background

Renal abscess is a rare but serious disease among children, and it represents the most severe form of infectious kidney disease [1]. Delayed diagnosis of renal abscesses commonly leads to poor therapeutic outcomes [2]. Extended-spectrum β-lactamase (ESBL) is a bacterial enzyme that causes resistance to various type of antibiotics, including third-generation cephalosporins and monobactams [3]. Increasing rates of ESBL-producing pathogens is an emerging worldwide problem because the community-acquired infections they cause are difficult to manage [3]. Here, we report the case of a previously healthy child who developed a renal abscess from bacteremia caused by community-acquired ESBL-producing *Escherichia coli*. We discuss the diagnostic difficulty and management of this condition.

## Case presentation

A 5-year-old girl presented to our department with high fever, headache, and vomiting for 2 days, and a high inflammatory response was found. She had commenced amoxicillin (40 mg/kg/day) a day before presentation. She never had a urinary tract infection (UTI) or a history of fever without a focus. Her general condition was good, and physical examination showed mild redness in the throat and no signs of Kawasaki disease. Laboratory tests revealed a high inflammatory response (white blood cell count [WBC], 21,080/μL [neutrophil, 85.8%]; C-reactive protein [CRP] level, 19.5 mg/dL), and a bacterial infection was suspected. Although no bacteria were identified in the urine on Gram staining, the urine test showed WBC > 100/high-power field, and we diagnosed her with a UTI. We administered both ampicillin (150 mg/kg/day) and cefotaxime (150 mg/kg/day) intravenously.

**Fig. 1:**
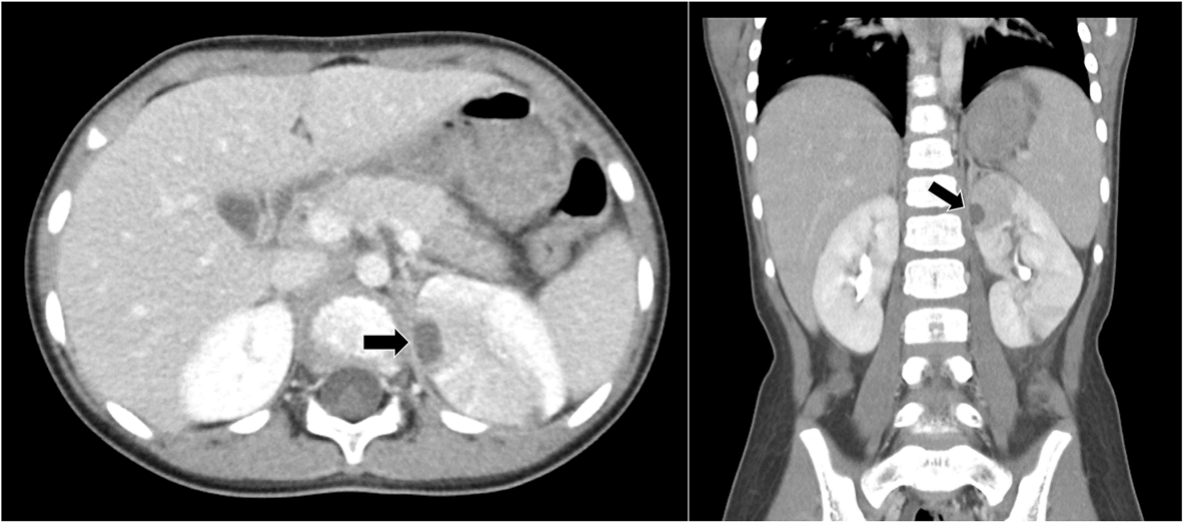
Computed tomography (CT) scan performed on day 3 of admission shows a left superior renal abscess (13 × 9 × 10 mm, arrow). Low-density areas in the superior and anterior poles of the left kidney suggesting acute focal bacterial nephritis are seen

A day after admission, gram-negative rods were detected from blood cultures. Although she continued to have a high fever, her general condition was good, and recurrent blood tests showed that the CRP levels had reduced to 14.1 mg/dL. We could not detect any signs of hydronephrosis, acute focal bacterial nephritis, or renal abscesses on renal ultrasound. We thought that the antibiotics were effective and therefore continued with the same treatment. However, the fever was prolonged, continuing on the third day of admission when contrast-enhanced computed tomography (CT) was performed, and signs of renal abscess in the left kidney were observed (Fig. 1). On the same day, blood cultures came back positive for ESBL-producing *E. coli*. We then changed the antibiotics to meropenem (120 mg/kg/day).

**Fig. 2:**
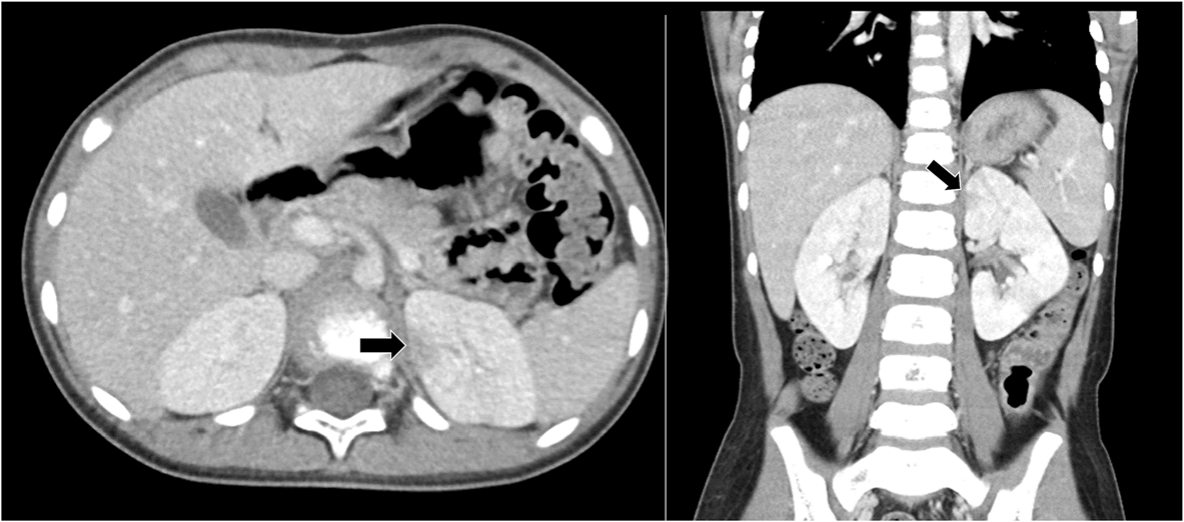
Repeat computed tomography (CT) scan after 3 weeks of meropenem administration. The renal abscess and poor contrast enhancement in the left kidney showed improvement, but poor contrast enhancement remained in in the area where the abscess was located

**Fig. 3:**
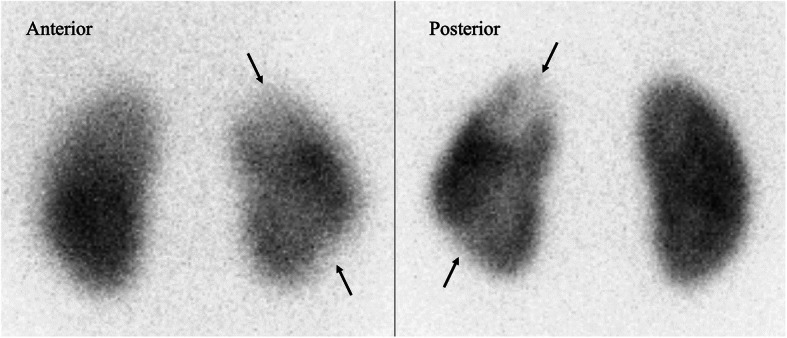
Dimercaptosuccinic acid (DMSA) scan obtained after 4 months from the onset of the disease. The decrease in the renal uptake of radionuclide in the upper and lower poles of the left kidney along the low-density areas in the initial CT scan suggested renal scarring (arrows)

We did not perform drainage of the renal abscess because the mass was only 10 mm and too small to puncture. ESBL-producing *E. coli* were also detected in the urine cultures obtained on admission. Her fever resolved on day 5, and there was no recurrent fever. After 3 weeks of treatment with meropenem, improvements of the left kidney abscess were observed on repeat CT scanning (Fig. 2). Although poor contrast enhancement remained in the area where the abscess was located, we did not perform another CT scan to check for further improvement to avoid exposing the patient to additional radiation. Technetium-99 m dimercaptosuccinic acid renal scanning conducted 4 months after the onset of symptoms revealed the presence of renal scarring in the upper and lower poles of the left kidney along the low-density areas in the initial CT scan (Fig. 3). No signs of vesicoureteral reflux were found with a voiding cystourethrogram, and no decrease in renal function was found on laboratory examination. At the 1-year follow-up, she had no recurrence of the abscess or renal sequelae such as hypertension, proteinuria, or impaired kidney function.

## Discussion and conclusion

Renal abscess is a rare bacterial infection among children. To our knowledge, this is the first report of a renal abscess in a pediatric patient with bacteremia caused by community-acquired ESBL-producing *E. coli*. Increasing rates of fecal carriage of ESBL-producing Enterobacteriaceae in children have been reported [4]. The occurrence of UTIs caused by these bacteria has also increased [5], and the selection of empirical treatments for UTI is becoming complicated. Only one case of pediatric renal abscess caused by community-acquired ESBL *E. coli* has previously been reported, and it had a favorable outcome without bacteremia [6].

Our patient developed a renal abscess caused by ESBL-producing *E. coli* despite previously being healthy. Several studies have reported that an underlying disease, recurrent UTI, or recent hospitalization are potential risk factors for UTI caused by ESBL-positive microorganisms [5, 7, 8]. In addition, prophylactic antibiotics, especially cephalosporins for urinary tract anomalies, have been reported as risk factors for UTIs caused by community-acquired ESBL-producing microorganisms [9, 10]. Although patients with a renal abscess often have urinary tract abnormalities such as vesicoureteral reflux, patients without underlying diseases can also be affected by renal abscesses [2].

In this case, the urine culture was positive after confirmation of a positive blood culture and no signs of vesicoureteral reflux were found. Although the etiology of the renal abscess was not fully understood, ascending infection due to vesicoureteral reflux and hematogenous dissemination are thought to be potential etiologies [2]. Renal abscess in the absence of a positive urine culture and no signs of vesicoureteral reflux may suggest an etiology of hematogenous dissemination. Physicians should be aware of false-negative cases of vesicoureteral reflux, such as a situation in which the bladder was not filled over the age-adjusted bladder capacity, although vesicoureteral reflux is usually used for evaluating voiding cystourethrogram [11].

Nausea, vomiting, abdominal pain, and flank pain are typical symptoms of a renal abscess [2]. However, in children, the absence of such a specific set of symptoms often makes it difficult to obtain an early diagnosis [2, 12]. Renal abscesses are usually confirmed by medical imaging, and CT scans are the preferred diagnostic modality, in part because they help diagnose the condition earlier than sonography [2]. Although the present case revealed vomiting before presentation, 2 days passed before the renal abscess was diagnosed from a CT scan.

Treatment for renal abscesses involves a combination of appropriate antibiotics and percutaneous or open surgical drainage. Only intravenous antibiotics were used in this case, because the abscess was too small to drain. In adult populations, it is recommended that renal abscesses less than 3 cm are treated without drainage, and this recommendation is also frequently applied to pediatric patients. Case studies in children have reported that renal abscesses less than 3 cm can be successfully treated without drainage [13, 14].

Although the preferred antibiotic for ESBL-positive microorganisms is carbapenem, the selection of other antibiotics is under discussion due to antimicrobial resistance. Carbapenem treatment needs to be given intravenously and requires a longer duration of hospital stay [15, 16]. Two previous retrospective studies showed that the initial empiric antibiotic therapy for pediatric UTIs caused by ESBL did not worsen the outcome, and the authors concluded that switching to appropriate antibiotics after detection of ESBL-producing microorganisms was sufficient [17, 18]. The efficacy of non-carbapenem antibiotics such as amikacin, fosfomycin, cefmetazole, and flomoxef for UTIs with ESBL-producing bacteria has been reported [9, 15, 19]. However, the efficacy of antibiotics other than carbapenem for blood culture-positive UTIs caused by ESBL-producing pathogens among children is unknown. Because the use of piperacillin-tazobactam as a “carbapenem-sparing” option was inferior to meropenem with bacteremia of an ESBL-producing pathogen in an adult population [20], selection of carbapenem-sparing antibiotics for similar cases should be carefully considered. Thus, there is an opinion that carbapenem be recommended from the beginning of treatment for renal abscesses in children to avoid worsening of the outcomes [1]. Regardless of a longer hospital stay, we continued meropenem for 3 weeks in the present case, which was blood culture positive with a renal abscess caused by ESBL-producing *E. coli*. We did not suspect an ESBL-producing pathogen infection at first and therefore treated the patient with non-carbapenem antibiotics before switching to carbapenem after confirming the results of the culture. Although we successfully treated the patient, it required us to investigate appropriate empiric and definitive therapies for a pediatric renal abscess with bacteremia caused by ESBL-positive microorganisms.

Preventing permanent renal scarring is one of the important goals in the management of UTIs, especially in patients with renal abscesses. As delayed antimicrobial treatment is associated with permanent renal scarring among children with febrile UTIs [21], early diagnosis and appropriate interventions are important for preventing severe complications. Although our patient developed renal scarring, we cannot conclude if this was caused by the infection or from a kidney malformation. It is also worth noting that renal scarring is not always related to long-term complications. Renal scars can cause long-term impaired kidney function, high blood pressure, and complications during pregnancy. Although there are heterogeneous reports about the long-term outcomes of renal scarring, mild to moderate renal scarring may correlate with few long-term complications [22]. Thus, patients with renal abscesses should be managed carefully with consideration of the harmful effect of studies, such as radiation exposure.

The present case had some limitations for considering appropriate management of the renal abscess. We cannot decide if carbapenem-sparing empiric therapy is appropriate for larger renal abscesses because the renal abscess in the present case was small. Thus, this study does not confirm the appropriateness of the continuation of sensitive non-carbapenem antibiotics.

Based on our study, we suggest that, for children with a clinical suspicion of renal abscesses, early imaging studies, especially CT scans, should be performed to prevent complications such as renal scarring. A previous study had described the importance of kidney ultrasound and nephromegaly findings as good indicators for suspecting renal abscess and performing CT scans [2]. In addition, earlier detection of sonographic-marked nephromegaly may lead to the diagnosis of acute focal bacterial nephritis before its progression into renal abscess [2]. Furthermore, the choice of carbapenem-sparing empiric antibiotics for renal abscesses with bacteremia caused by ESBL-producing *E. coli* was deemed appropriate in this case. Owing to the lack of evidence regarding pediatric renal abscesses caused by ESBL-producing pathogens, empirically broader antibiotics, such as carbapenem, should also be considered until antimicrobial susceptibility is confirmed.

Given the increasing prevalence of ESBL-producing microorganisms, clinicians should be cognizant of the possibility of renal abscesses caused by these microorganisms, even among previously healthy children, when examining fevers of unknown origin. Once a renal abscess is suspected, early diagnosis and management are important to reduce the risk of life-threatening complications and renal scarring. Although we successfully managed the condition with carbapenem-sparing antibiotic treatment in this case initially, it required us to investigate appropriate therapies for pediatric renal abscesses with bacteremia caused by ESBL-positive microorganisms.

## Data Availability

Not applicable.

## Abbreviations

CRP: C-reactive protein
CT: computed tomography
*E. coli*: *Escherichia coli*
ESBL: Extended-spectrum β-lactamase
UTI: Urinary tract infection
WBC: White blood cell count
